# A Nomogram for Predicting Pulmonary Embolism in Silicosis Patients

**DOI:** 10.1111/crj.70059

**Published:** 2025-03-04

**Authors:** Jiaqing Zhou, Wen Du, Jin Liu, Lijun Peng

**Affiliations:** ^1^ Department of Respiratory Medicine, West China School of Public Health and West China Fourth Hospital Sichuan University Chengdu China; ^2^ Department of Epidemiology, School of Public Health Fudan University Shanghai China

**Keywords:** CTPA, nomogram, Padua score, silicosis

## Abstract

**Background:**

As one of the most severe occupational diseases that prevention efforts have supported for several decades, silicosis is still a public health issue that lacks a prediction model for pulmonary embolism.

**Methods:**

A total of 162 patients confirmed to have silicosis were all involved in a training cohort to construct a nomogram with the outcome diagnosed by the CTPA using logistic regression. Univariate and LASSO analyses were used to select variables for the nomogram.

**Result:**

mMRC, pectoralgia, history of VTE, active tumor, unilateral lower limb pain or edema, hormonotherapy, reduced mobility, and heart failure/respiratory failure were selected for the establishment of the nomogram for silicosis with pulmonary embolism.

**Conclusion:**

A novel nomogram was developed to predict pulmonary embolism in silicosis patients. The internal validation indicated that clinicians could utilize this predictive model to help decision‐making and patient management.

## Graphical abstract

This is a data‐driven and evidence‐based study to better understand the risk factors for silicosis patients with pulmonary thromboembolism. Nomogram‐based predictive models were developed and validated to forecast pulmonary thromboembolism probability for silicosis patients. The prediction effect of the Padua score on pulmonary thromboembolism was compared with that of the model established in this study

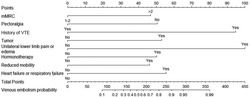

## 1 Introduction

Although efforts have been taken to prevent the global occupational hazard for decades, pneumoconiosis is still a significant illness for coal workers, building workers, and workers in constant contact with silica dust worldly [1, 2]. As a common disease worldwide, the epidemic of pneumoconiosis in China is serious particularly, which resulted from rapid development and less developed institutions, causing millions of workers to be exposed to occupational dust hazards [3]. And pneumoconiosis is a major challenge in China, which accounts for over 90% of the reported occupational diseases patients, and the tendency of high‐proportion pneumoconiosis is consistent in other undeveloped countries [4, 5, 6]. Including a group of severe lung damage associated with the occupational inhalation of dust and corresponding reactions of lung tissues, silicosis can eventually proceed to progressive disabilities accompanied by pulmonary tuberculosis and lung cancer [7, 8, 9]. Pulmonary embolism (PE) is a severe pneumoconiosis complication and a type of venous thromboembolism (VTE), and the mortality rate in untreated cases reaches around 30% [10, 11, 12, 13]. The diagnostic work‐up of suspected pulmonary embolism includes the application of disease history, clinical symptoms, and D‐dimer testing; however, pulmonary embolism confirmation is notoriously tricky to diagnose clinically [13, 14]. For patients with a high risk of pulmonary embolism, CT pulmonary angiography (CTPA) can find and define emboli to the level of segmental pulmonary arteries; therefore, it shows more accuracy than other detection like sonography and is used increasingly in clinical work as the golden standard of diagnosis [14, 15].

Only patients at high risk of pulmonary embolism are recommended to receive CTPA due to high inspection costs. The Padua prediction score is a risk assessment model for identifying high‐risk groups of embolism, showing good prediction power in previous studies [16, 17]. However, the Padua score consists of lengthy and jumbled items, causing complex valuation in clinical applications and the insufficiency of accuracy sometimes. Presenting individualized prediction in simplified segments and scores, the nomogram is a graphic model based on a mathematical formula from kinds of the regression model and has been widely used in the prediction of prognosis or disease state in the past decade [18, 19].

In this study, we aimed to develop a new nomogram model for the prediction of pulmonary embolism in silicosis that provides the personalized probability of embolism rather than a risk stratification and compare the model discrimination with the traditional Padua prediction score, using AUC and decision curve analysis (DCA), to improve diagnosability, reduce unnecessary use of medical resources, and provide a reference for clinical decision‐making.

## 2 Methods

### 2.1 Patients and Data Selection

**FIGURE 1:**
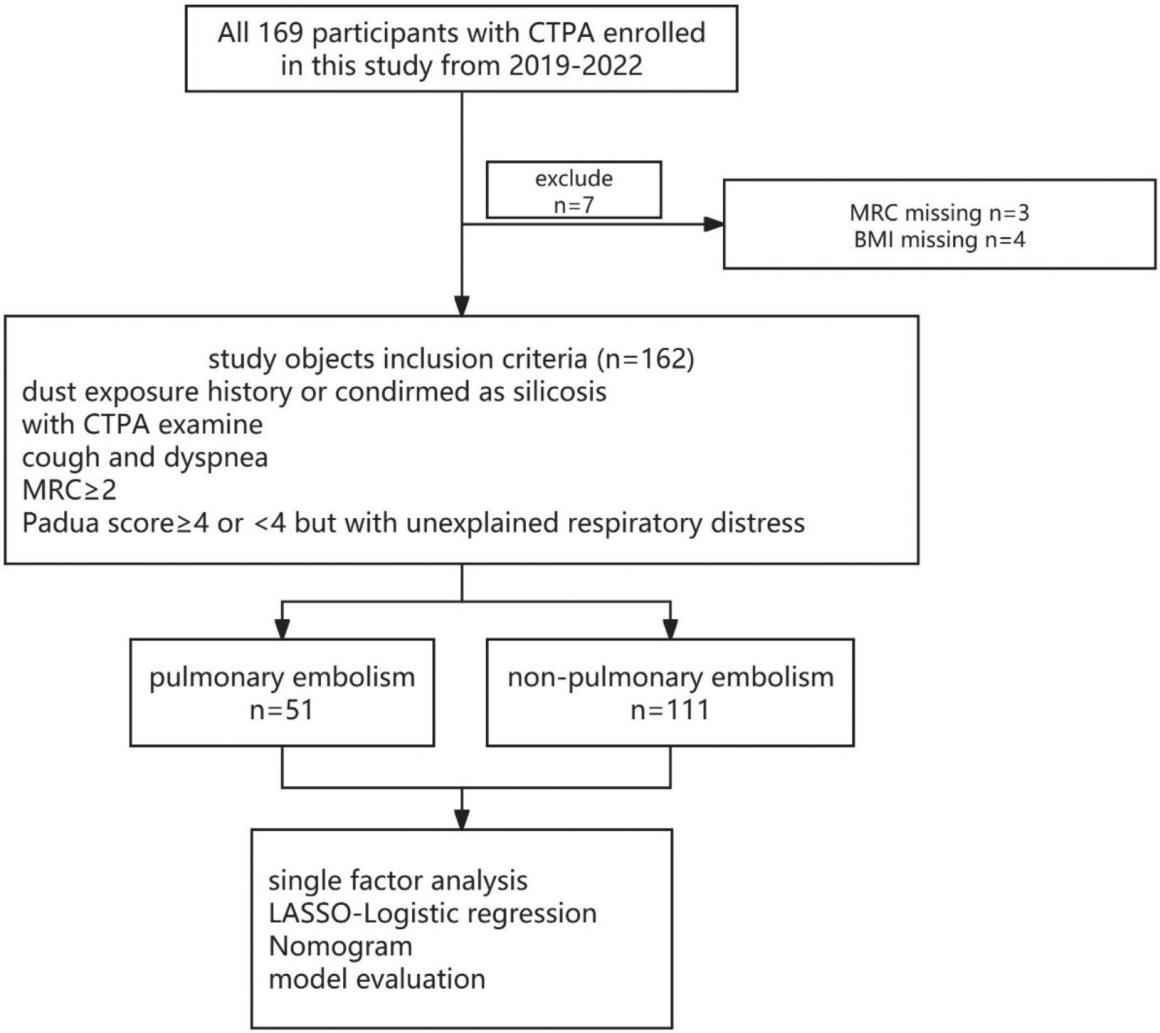
Flowchart for study design.

Retrospective and prospective collections were both involved in this study. All patients diagnosed with silicosis accepting treatment in this hospital from 2019 to 2022 were included in the research. Each participant was assigned informed consent for medical research, and an authorization approved the study of ethical review from the West China School of Public Health and West China Fourth Hospital, Sichuan University. The exclusion criteria were information missing (characteristic), rejection to be followed up, without CTPA examination. Individuals with a history of past dust exposure due to occupational activities, or clinically and diagnostically confirmed as silicosis, based on the diagnosis of occupational pneumoconiosis (GBZ70‐2015 in China), and in accordance with the consensus of experts on standardized chest CT examination techniques for pneumoconiosis (2020 edition). Additionally, patients must undergo CTPA examination, which necessitates meeting the following conditions beyond a history of dust exposure or a diagnosis of silicosis: presenting with symptoms such as cough and dyspnea after activity, recent exacerbation of symptoms with an MRC score of 2 or above; undergoing thrombotic risk assessment upon admission, with a Padua score greater than 4 or less than 4, but with unexplained respiratory distress clinically suggestive of pulmonary embolism (shown in Figure 1).

The silicosis patients were diagnosed with occupational exposure history, self‐reported symptoms, and laboratory and imaging results. Demographic data with dust exposure history were collected during the first admission by disease history inquiry. Other data were from electronic medical record information, and participants did not be followed up after hospital discharge. Identification of pulmonary venous embolism was according to the result of CTPA at admission. The Padua score stratifies low‐risk (< 4) and high‐risk groups (≥ 4), and the latter group was recommended to receive a CTPA examination to confirm the embolism and treatment with preventive measures to induce adverse events [16]. We collected age (≥ 65 and < 65), gender, stage, exposure time, BNP (pg/mL), course of the disease, mMRC index (modified British Medical Research Council index), tuberculosis history, cough, polypnea, pectoralgia, hemoptysis, D‐dimer (mg/L), heart rate, electrocardiogram, lower‐extremity sonography, cardiac sonography, history of VTE, surgery or trauma within 1 month, tumor, unilateral lower limb pain or edema, hormonotherapy, acute infection, heart failure or respiratory failure, diabetes history, Padua prediction score, and CTPA. In this study, we defined the high‐risk group by Padua as patients with embolism.

### 2.2 Statistical Analysis

For continuous variables, we used the mean ($\bar{x}$) and standard deviation (SD) to describe the centralized tendency and dispersion degree, which applied to a normal distribution, while we used the median and IQR (interquartile range) for those who did not follow the normal distribution. Most variables in this study were categorical variables, and frequency and percentages were presented to describe the characteristics of patients. Dusting time was defined as the years of overall dust‐catching, and patients may had a discontinuous time of exposure to dust.

For the primary data exploration and presentation, we used the *t*‐test, Kruskal–Wallis test, and chi‐square test to compare the difference between the embolism and embolism‐free groups by CPTA. The LASSO‐based analysis was employed to select variables for modeling. Furthermore, selected potential risk factors were included in the multivariate logistic regression to build a model, which was the base of the nomogram. After the nomogram development, the estimation of AUC (95% CI) and the plotting of calibration curves were both to evaluate the discrimination and consistency of the prediction model. Model internal validation was included in calibration plots with the bootstrap method (B = 1000). DCA analysis, the net reclassification index (NRI), and integrated discrimination improvement (IDI) presented the prediction performance and clinical benefits difference between the new model and the Padua score [20, 21]. All *p* values in this study were two‐tailed, and the *p* less than 0.05 was defined as statistical significance. R software (4.2.1) was utilized in all analyses, tabling, and mapping.

## 3 Results

### 3.1 Demographic and Clinical Characteristics

A total of 162 patients were identified with silicosis, and all were included to the modeling. The demographic, clinical, and laboratory characteristics of these patients are summarized in Table 1. The average age of the two groups had no statistical difference, while the embolism patients were slightly older than those without, and the mean age of all patients was around 58. Of all patients, approximately a seventh‐eighth of individuals were male (86.5%), but males had not a higher embolism rate than females. Stage III patients accounted for more than two‐thirds of patients (72.8%), and the two groups did not show a difference in the stage. Patients with longer time exposure to dust were associated with higher embolism rates (*p* = 0.035). Around one‐third patients were abnormal BNP (≥ 125 pg/mL), and the embolism group had a higher positive rate (*p* = 0.015). The course of the disease was longer in the embolism group (*p* = 0.085). And the embolism group had a higher score. Tuberculosis history was noted in 108 patients (66.7%), and proximally, all patients experienced cough and polypnea. For D‐dimer, the abnormal proportion of the embolism group (52.9%) was more than another group (*p* = 0.011). Less than half of the patients had no abnormalities in electrocardiogram findings, while the embolism group showed a lower proportion of normality. Nine participants had a history of VTE, but eight of them were funded by new embolisms with CPTA, demonstrating that recent pulmonary embolism was associated with VTE history. Unilateral lower limb pain or edema, reduced mobility, and heart and/or respiratory failure were differently distributed in the two groups (*p* < 0.05).

**TABLE 1:** Demographic and clinical features of participants by pulmonary embolism.

Variable	All (*n* = 162)	Embolism		*p*
		No (*n* = 111)	Yes (*n* = 51)	
Age	56.8 (9.46)	56.3 (9.25)	57.9 (9.93)	0.343
Gender				0.998
Male	140 (86.4%)	96 (86.5%)	44 (86.3%)	
Female	22 (13.6%)	15 (13.5%)	7 (13.7%)	
BMI (mean, SD)	22.1 (1.12)	22.3 (1.45)	21.8 (2.14)	0.382
Different stages of pneumoconiosis				0.698
I	23 (14.2%)	16 (14.4%)	7 (13.7%)	
II	21 (13.0%)	16 (14.4%)	5 (9.80%)	
III	118 (72.8%)	79 (71.2%)	39 (76.5%)	
Dust exposure time (year)	10.00 (4.00)	10.00 (10.00)	11.00 (12.00)	0.036
0–10	101 (62.3%)	76 (68.5%)	25 (49.0%)	
> 10	61 (37.7%)	35 (31.5%)	26 (51.0%)	
BNP (pg/mL)				**0.015**
0–125	97 (59.9%)	74 (66.7%)	23 (45.1%)	
≥ 125	65 (40.1%)	37 (33.3%)	28 (54.9%)	
Course of the disease (year)	9.00 (600)	8.00 (7.00)	10.00 (4.25)	0.085
mMRC				**< 0.001**
1	7 (4.32%)	7 (6.31%)	0 (0.00%)	
2	55 (34.0%)	47 (42.3%)	8 (15.7%)	
3	74 (45.7%)	44 (39.6%)	30 (58.8%)	
4	26 (16.0%)	13 (11.7%)	13 (25.5%)	
Tuberculosis				0.590
No	54 (33.3%)	35 (31.5%)	19 (37.3%)	
Yes	108 (66.7%)	76 (68.5%)	32 (62.7%)	
Cough				**0.022**
No	8 (4.94%)	2 (1.80%)	6 (11.8%)	
Yes	154 (95.1%)	109 (98.2%)	45 (88.2%)	
Polypnea				0.679
No	7 (4.32%)	4 (3.60%)	3 (5.88%)	
Yes	155 (95.7%)	107 (96.4%)	48 (94.1%)	
Pectoralgia				0.504
No	130 (80.2%)	87 (78.4%)	43 (84.3%)	
Yes	32 (19.8%)	24 (21.6%)	8 (15.7%)	
Hemoptysis				0.635
No	126 (77.8%)	88 (79.3%)	38 (74.5%)	
Yes	36 (22.2%)	23 (20.7%)	13 (25.5%)	
D‐dimer (mg/L)				**0.011**
< 0.55	101 (62.3%)	77 (69.4%)	24 (47.1%)	
≥ 0.55	61 (37.7%)	34 (30.6%)	27 (52.9%)	
Electrocardiogram				**0.047**
Normal	74 (45.7%)	56 (50.5%)	18 (35.3%)	
Right heart	43 (26.5%)	22 (19.8%)	21 (41.2%)	
Sinus tachycardia	34 (21.0%)	25 (22.5%)	9 (17.6%)	
Other	11 (6.79%)	8 (7.21%)	3 (5.88%)	
Lower‐extremity sonography				**0.019**
Normal	147 (90.7%)	105 (94.6%)	42 (82.4%)	
Thrombus	15 (9.26%)	6 (5.41%)	9 (17.6%)	
Cardiac sonography				1
Normal	90 (55.6%)	62 (55.9%)	28 (54.9%)	
Right heart	72 (44.4%)	49 (44.1%)	23 (45.1%)	
History of VTE				**< 0.001**
No	153 (94.4%)	110 (99.1%)	43 (84.3%)	
Yes	9 (5.56%)	1 (0.90%)	8 (15.7%)	
Surgery or fracture within 1 month				0.098
No	160 (98.8%)	111 (100%)	49 (96.1%)	
Yes	2 (1.23%)	0 (0.00%)	2 (3.92%)	
Tumor				**0.012**
No	152 (93.8%)	108 (97.3%)	44 (86.3%)	
Yes	10 (6.17%)	3 (2.70%)	7 (13.7%)	
Heart rate (bpm)				0.489
≤ 75	28 (17.3%)	19 (17.1%)	9 (17.6%)	
76–94	58 (35.8%)	43 (38.7%)	15 (29.4%)	
≥ 95	76 (46.9%)	49 (44.1%)	27 (52.9%)	
Unilateral lower limb pain or edema				**< 0.001**
No	149 (92.0%)	109 (98.2%)	40 (78.4%)	
Yes	13 (8.02%)	2 (1.80%)	11 (21.6%)	
Hormonotherapy				**0.004**
No	94 (58.1%)	73 (56.9%)	21 (41.2%)	
Yes	68 (41.9%)	38 (34.2%)	30 (58.8%)	
Acute infection				0.778
No	15 (9.26%)	11 (9.91%)	4 (7.84%)	
Yes	147 (90.7%)	100 (90.1%)	47 (92.2%)	
Reduced mobility				**< 0.001**
No	148 (91.4%)	109 (98.2%)	39 (76.5%)	
Yes	14 (8.64%)	2 (1.80%)	12 (23.5%)	
Heart failure or respiratory failure				**< 0.001**
No	87 (53.7%)	74 (66.7%)	13 (25.5%)	
Yes	75 (46.3%)	37 (33.3%)	38 (74.5%)	
DM				0.138
No	148 (91.4%)	104 (93.7%)	44 (86.3%)	
Yes	14 (8.64%)	7 (6.31%)	7 (13.7%)	
Padua score	2.72 (1.70)	2.23 (1.16)	3.78 (2.15)	**< 0.001**

*Note:* The *p*‐values (in bold) correspond to statistical tests that compare the demographic and clinical characteristics of the two groups of patients, namely, those with thrombosis and those without.

### 3.2 Predictors Selection and Nomogram Construction

**FIGURE 2:**
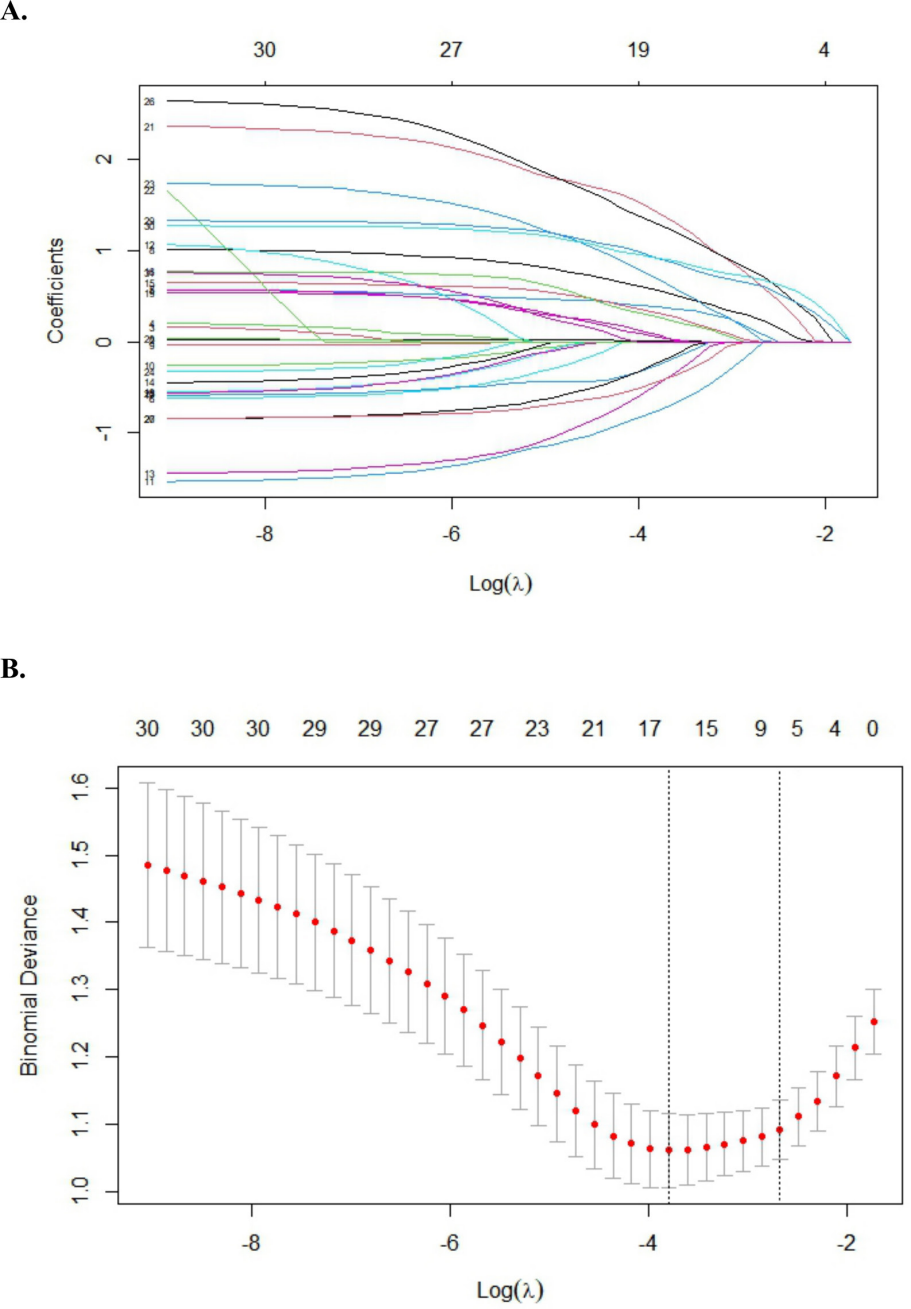
Feature selection using the LASSO binary logistic regression model. (A) LASSO coefficient profiles of the baseline features. (B) Tuning parameter (λ) selection in the LASSO model used 10‐fold cross‐testing via minimum criteria.

All variables measured at the baseline were included in LASSO regression, and the variation characteristics of the coefficient of all variables were shown in Figure 2A. After screening, the following eight variables remained significant predictors of pulmonary embolism: mMRC, pectoralgia, history of VTE, tumor, unilateral lower limb pain or edema, hormonotherapy, reduced mobility, and heart failure or respiratory failure.

**FIGURE 3:**
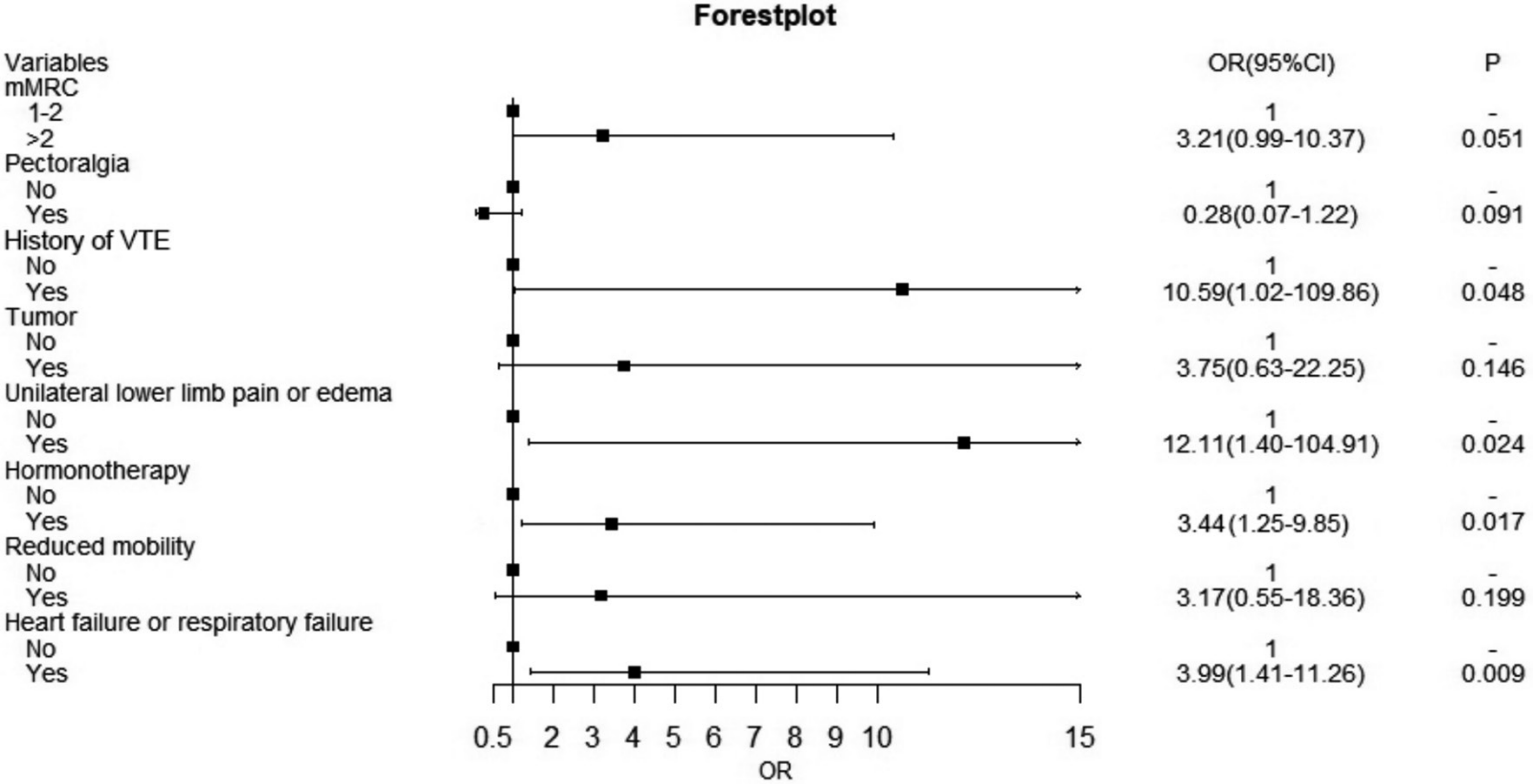
Forest plot for selected variables of the multivariate logistic regression.

**FIGURE 4:**
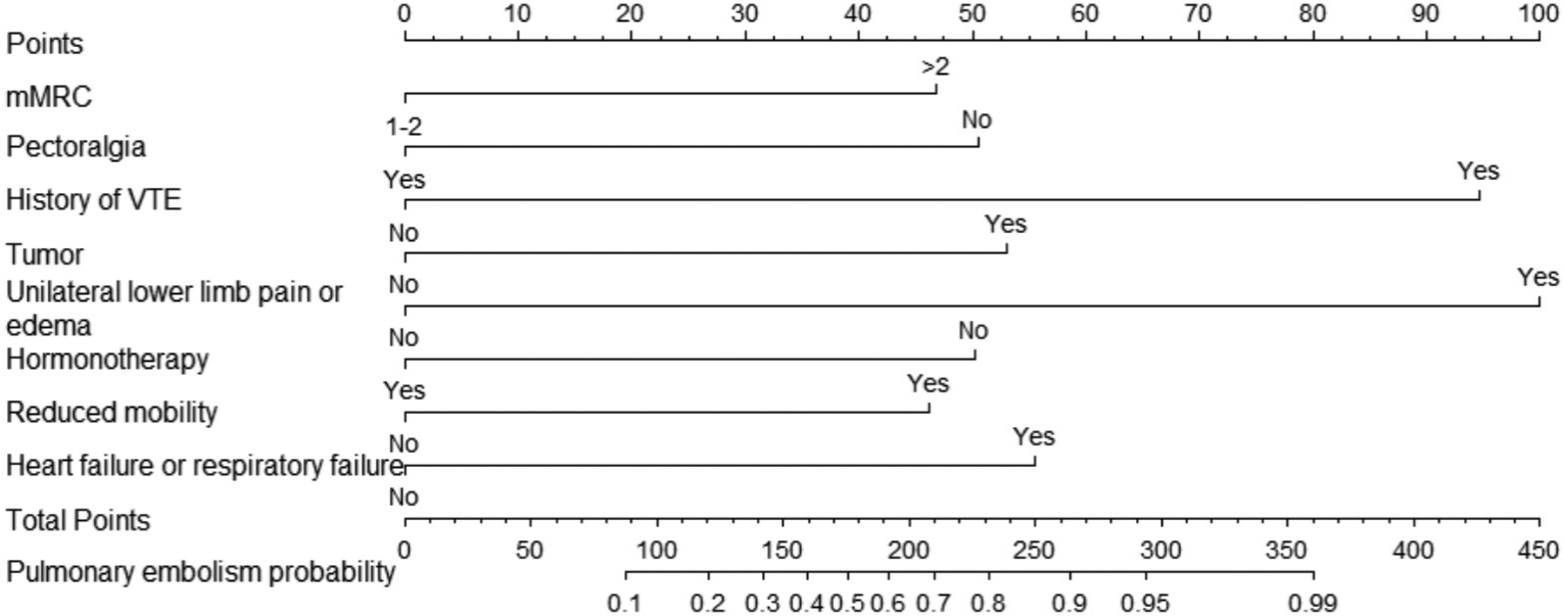
Nomogram based on the LASSO–logistic regression.

**FIGURE 5:**
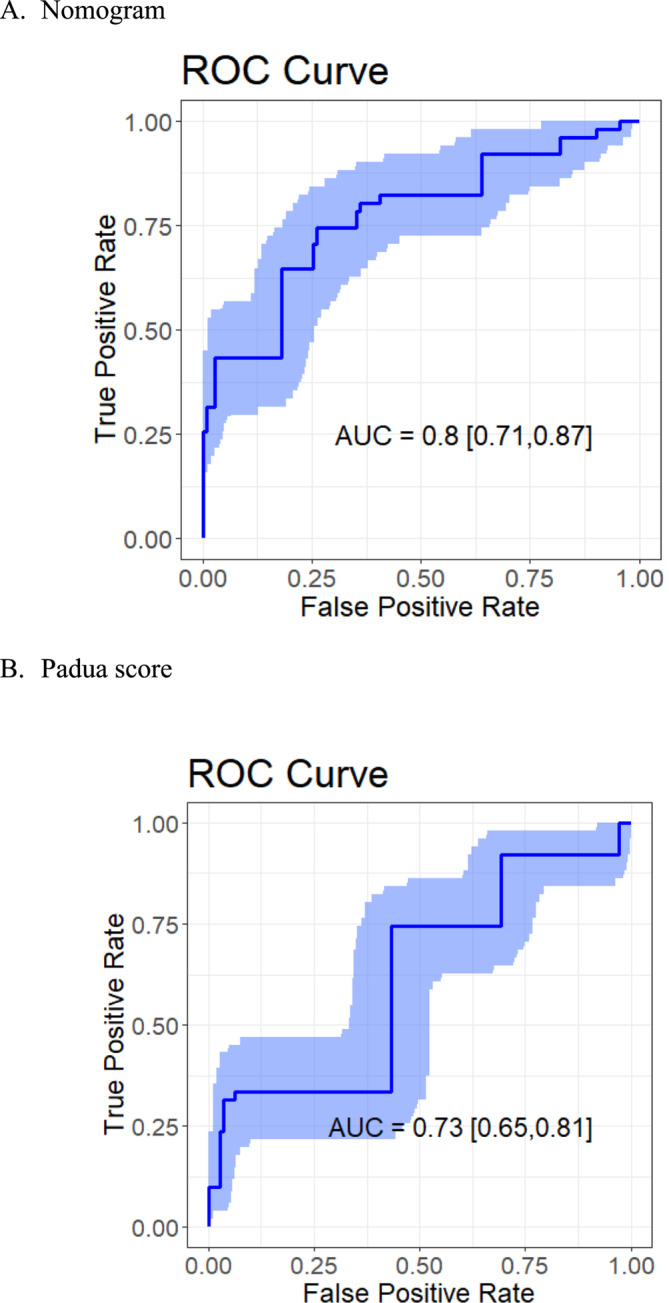
ROC curves for nomogram and Padua score using the current database.

**FIGURE 6:**
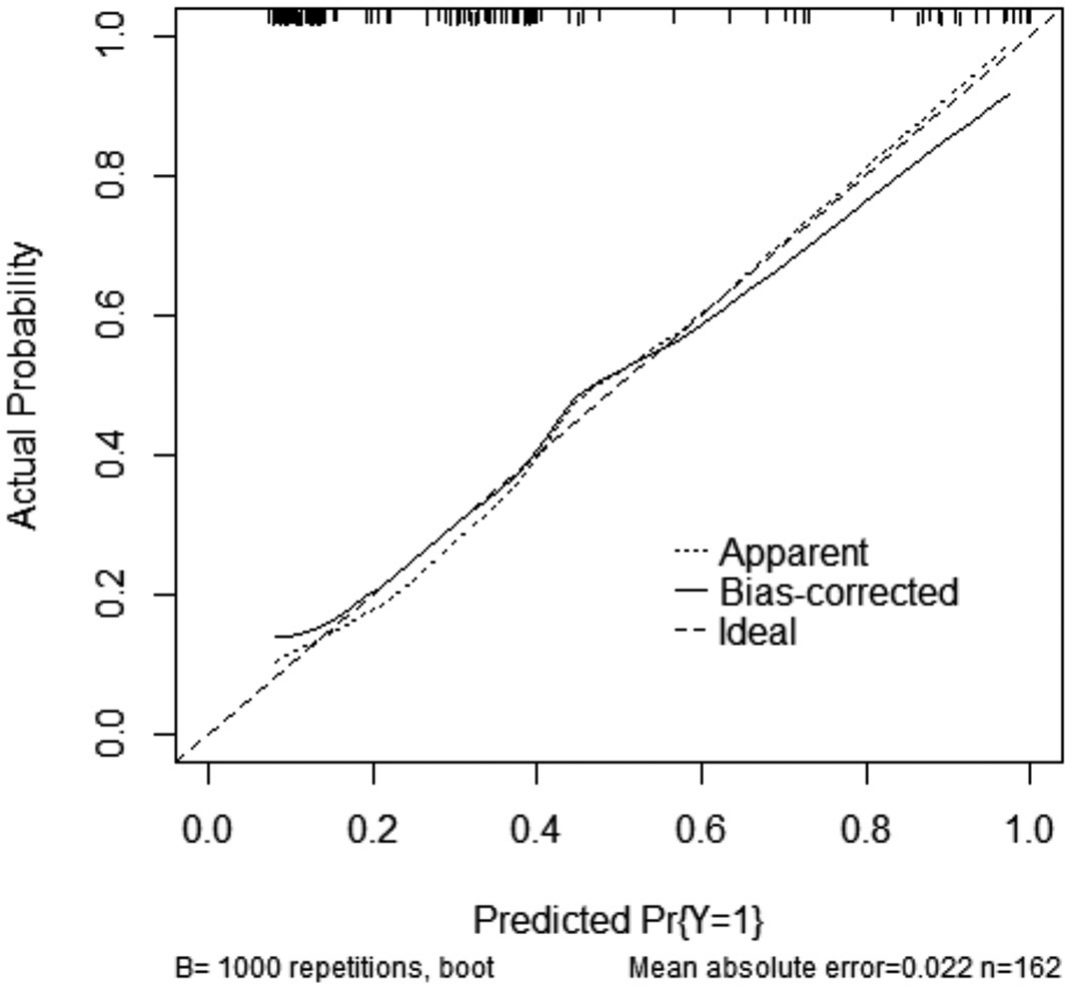
Calibration curve of nomogram.

**FIGURE 7:**
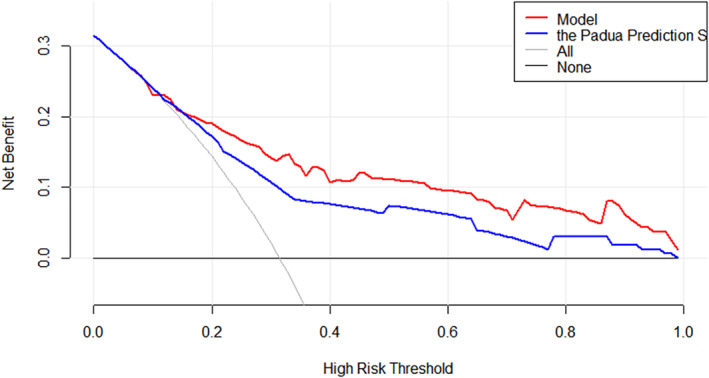
DCA curves analysis of the nomogram and Padua score.

Furthermore, multivariate logistic regression was based on these eight selected variables. Figure 3 presented the ORs and 95% CIs for the selected variables in the logistic model. Heart or respiratory failure was associated with embolism independently. Based on the LASSO–logistic regression, the nomogram model to predict pulmonary embolism with pneumoconiosis was shown in Figure 4. The AUC was 0.80 (95% CI: 0.71–0.87, *p* < 0.001) and 0.73 (95% CI: 0.65–0.81, *p* < 0.001) for the nomogram and Padua score, respectively (Figure 5). *Z* test reflected that AUCs between the two prediction models were statistically different (*p* = 0.002). The calibration curve based on the bootstrap method (B = 1000) showed that the prediction probabilities of the new model were close to the actual observations, the prediction curve fit the diagonal line well, and the Hosmer–Lemeshow test contained *p* values of 0.285. (Figure 6) Additionally, we further conducted the DCA analysis to assess the clinical performance of the new model and the Padua score by plotting two DCA curves in one map (Figure 7), with the NRI [0.223 (95% CI: 0.066–0.382, *p* = 0.005)] and the IDI [0.111 (95% CI: 0.042–0.180, *p* = 0.002)]. (Figure 7).

## 4 Discussion

Globally, the top three incidents of silicosis were found in Europe, China, and the United States, and the prevalence in developing regions was much higher [22]. Silicosis accounts for almost 50% of pneumoconiosis patients and remains one of the most severe occupational diseases in China [23]. The course of pneumoconiosis can last for a decade or even decades, and it requires long‐term inhalation of dust to cause structural changes in the lungs. As a risk factor for pulmonary venous embolism, the symptoms of pneumoconiosis can cover up the clinical manifestations of pulmonary embolism and aggravate the progress of the disease [11, 24]. Accurate information on embolism diagnosis is important for clinical decisions and management, and widely used prediction systems like the Padua score include complex variables but lack flexibility and intuition in making individual prognostication. Therefore, the goal of this study was to demonstrate the risk factors that can be utilized for early screening of pulmonary embolism. The results showed that the multiple variables logistic model based on LASSO regression was able to better predict the risk of pulmonary embolism in pneumoconiosis patients, compared with the Padua score alone. Furthermore, a novel nomogram was developed for the visualization of the prediction model as an auxiliary tool for clinicians.

A total of 162 patients were included in this study. Eight factors were selected according to the LASSO regression results, VTE history, unilateral lower limb pain or edema, hormonotherapy, and heart failure or respiratory failure were independent influence factors for pulmonary embolism in coal workers' pneumoconiosis. Breathless patients with mMRC grade more than 2 were more likely to have embolism (*p* < 0.05 in univariate analysis, *p* = 0.051 in multivariate logistic regression, OR [95% CI]: 3.21 [0.99, 10.37]). Although after adjustment of other variables, the association between mMRC and pulmonary embolism was not statistically significant, prior studies noted its importance. The mMRC dyspnea scale was recommended by guidelines in many countries to make a measurement of breathlessness severity and was often the endpoint of clinical trials to evaluate treatment effects [25]. The current study found that the history of VTE was also a risk factor, with an odds ratio of 10.59 (*p* = 0.048), meaning that patients with a VTE history had a 10.59 times probability of experiencing pulmonary embolism versus those without a VET history. The reasons and pathophysiology contributing to VTE formation may be due to chronic inflammation, and most previous studies have considered deep venous thrombosis (DVT) caused the pulmonary embolism [11, 26]. However, a study conducted in Taiwan observed that pulmonary embolism was more frequent in pneumoconiosis, while DVT had twice incidence of pulmonary embolism in a general setting [11]. For this, previous studies proposed a hypothesis that pulmonary embolism originating de novo (DNPE) may be associated with pneumoconiosis [11, 27]. DNPE presented in patients with injury or inflammation as in previous investigations, and the thrombus formation may be promoted within pulmonary arteries instead of the peripheral venous system in pneumoconiosis patients due to perivascular fibrosis and muscularization [28, 29, 30]. Furthermore, the vascular change affected the ventilated blood flow, resulting in vortex and blood flow stasis, which caused platelet aggregation and promoted local thrombosis in the peripheral veins [31]. Therefore, DNPE may result from a similar response in pulmonary arteries. The active tumor was one of the strong provoking risk factors for venous thromboembolism, accounting for approximately 20% of all venous thromboembolism [32]. While in this study, this factor did not show a significant effect in the multivariate model (*p* = 0.146) but had a significant difference in proportion between pneumoconiosis patients with pulmonary embolism and those without. In this study, unilateral lower limb pain or edema was also a risk factor and was given a relatively higher score than other variables (due to a higher OR estimator of 12.11 [*p* = 0.024]). Additionally, as scoring items in the Padua score system, ongoing hormonal treatment, reduced mobility, and heart failure or respiratory failure were all included in the model, and all kept statistical significance for prediction except for reduced mobility.

Based on the selected factors, we developed a multivariate Logistic regression for evaluating the probability of pulmonary embolism in silicosis patients. And the nomogram showed higher accuracy and prediction power than a traditional system, the Padua score, with the AUC of 0.84 [95% CI: 0.76–0.90] for the nomogram and 0.73 [95% CI: 0.65–0.80] for the Padua in current study data (with significant difference between two AUC values). Besides, the nomogram presented an individual‐level risk assessment with simple and intuitive segments, being expected to help make clinical decisions. However, although the nomogram performed in prediction and passed the internal validation by the bootstrap method, external data were needed for external validation. Another limitation of this study is the small sample size. We cannot deny that the prediction model needs a big enough sample size from multiple centers to ensure the population presentation and external consistency.

Taken together, we established a nomogram based on the LASSO–logistic model with risk factors, which provided an individual forecast of the risk probability of pulmonary embolism in silicosis patients.

## Author Contributions

**Jiaqing Zhou:** conceptualization, methodology, data curation, formal analysis, writing – original draft, visualization. **Wen Du:** conceptualization, methodology, data curation. **Jin Liu:** conceptualization, methodology, formal analysis. **Lijun Peng:** supervision, writing – review & editing.

## Ethical Statement

The authors have nothing to report.

## Consent

The authors have nothing to report.

## Conflicts of Interest

The authors declare no conflicts of interest.

## Data Availability

The data that support the findings of this study are available on request from the corresponding author. The data are not publicly available due to privacy or ethical restrictions.
